# MRPS23 is a novel prognostic biomarker and promotes glioma progression

**DOI:** 10.18632/aging.205493

**Published:** 2024-01-31

**Authors:** Qiang Wang, Guiqing Chen, Liang Liu, Xiaoying Peng, Xian Yang, Ling Yang, Chunhong Li

**Affiliations:** 1 Department of Gastrointestinal Surgery, Suining Central Hospital, Suining 629000, Sichuan, P.R. China; 2 Department of Gynaecology, Suining Central Hospital, Suining 629000, Sichuan, P.R. China; 3 Department of Oncology, Suining Central Hospital, Suining 629000, Sichuan, P.R. China; 4 Department of Pathology, Suining Central Hospital, Suining 629000, Sichuan, P.R. China

**Keywords:** MRPS23, cell growth, glioma, prognostic, cell migration

## Abstract

Mitochondrial ribosomal protein S23 (MRPS23), a component of the ribosome small subunit, has been reported to be overexpressed in various cancers and has been predicted to be involved in increased cell proliferation. It has been confirmed that MRPS23 was involved in the regulation of breast cancer and hepatocellular carcinoma cell proliferation. However, little is known about the function of MRPS23 in glioma. In this study, we found that MRPS23 expression was higher in gliomas than in adjacent normal tissues. Higher expression of MRPS23 in gliomas correlated with poorer prognosis, unfavorable histological features, absence of mutations in the isocitrate dehydrogenase gene (IDH), absence of chromosome 1p and 19q deletions, and responses to chemoradiotherapy. Univariate and multivariate Cox analysis demonstrated MRPS23 expression was independently prognostic of overall survival, disease-free survival, and progression-free survival in patients with glioma. KEGG enrichment analysis results indicated that high MRPS23 expression was associated with cell proliferation and immune response-related signaling pathways. We also confirmed that MRPS23 was highly expressed in glioma cells lines, and MRPS23 knockdown significantly reduced cell survival, proliferation, and migration of glioma cells lines. Collectively, these findings offer mechanistic insights into how MRPS23 during glioma progression, and identify MRPS23 as a potential therapeutic target in the future.

## INTRODUCTION

Cancer is a multiple malignant disease in the world [1]. The malignant tumor patient prognosis is still poor [2]. In recent years, the number of tumor patients is gradually increasing, and the effectiveness of treatment is not ideal [3]. Therefore, the development of effective and specific tumor markers is extremely urgent for the diagnosis and treatment of tumors.

Mitochondrial ribosomal protein S23 (MRPS23), a component of the ribosome small subunit, has been reported to be overexpressed in various cancers and has been predicted to be involved in increased cell proliferation [4]. However, the MRPS23 role in pan-cancer diagnosis, prognosis, stem cell, RNA modification, and immune regulation remains unclear.

Our study utilized the MRPS23 role in human diverse cancer, and we clarified the association between MRPS23 expression and stem cell, RNA modification regulators expression, in human cancer. In short, MRPS23 is not only a potential prognosis biomarker in cancer, but also a promising therapeutic target for glioma.

## MATERIALS AND METHODS

### The MRPS23 expression in human cancer

The TIMER [5], TCGA, GTEx database and UALCAN database were used to examine the expression of MRPS23 in pan-cancer tissue [6].

### The prognosis and clinical information of MRPS23 in pan-cancer

The GEPIA databases and PrognoScan databases [7] were employed to analysis the OS, DSS and PFI of MRPS23 in pan-cancer. The MRPS23 gene mutation information was analyzed via cBioPortal [8].

### The relationship between stem cell and RNA modification of MRPS23 in pan-cancer

We downloaded MRPS23 gene expression and clinical data from UCSC (https://xenabrowser.net/) [9]. We employed the GeneMANIA and STRING database to construct gene–protein interactions network of MRPS23.

### Immunological functions analysis

The TIMER and XCELL tools were used to employ the MRPS23 immunological roles [10]. We adopted the TISIDB to detach the relationship between MRPS23 expression and immune modulator, obtained TMB, and MSI scores from TCGA.

### Cell lines and cell culture

Cell lines were purchased from the American Type Culture Collection (ATCC, Manassas, VA, USA) and cultured in DMEM medium supplemented with 10% FBS. All the cells were cultured at 37° C with 5% CO_2_. MRPS23 siRNA kits (si-MRPS23#1: 5’-GUGUAUGGGUCUGGUCAAATT-3’; si-MRPS23#2: 5’-GAAAUCCGAACACUUGAGUTT-3’) and negative control siRNAs (si-NC: 5’-UUCUCCGAACGUGUCACG UTT-3’) were purchased from GenePharma (China).

### CCK8 assays

For CCK8 assay, U87 and U251 cells were cultured in a 96-well plate (2,000 cells per well) supplemented with 200 μL of RPMI-1640 medium containing 10% FBS for 24, 48, 72 and 96 h, and measured with a CCK8 kit (Promega, Madison, WI, USA).

## RESULTS

### MRPS23 expression in pan-cancer

**Figure 1:**
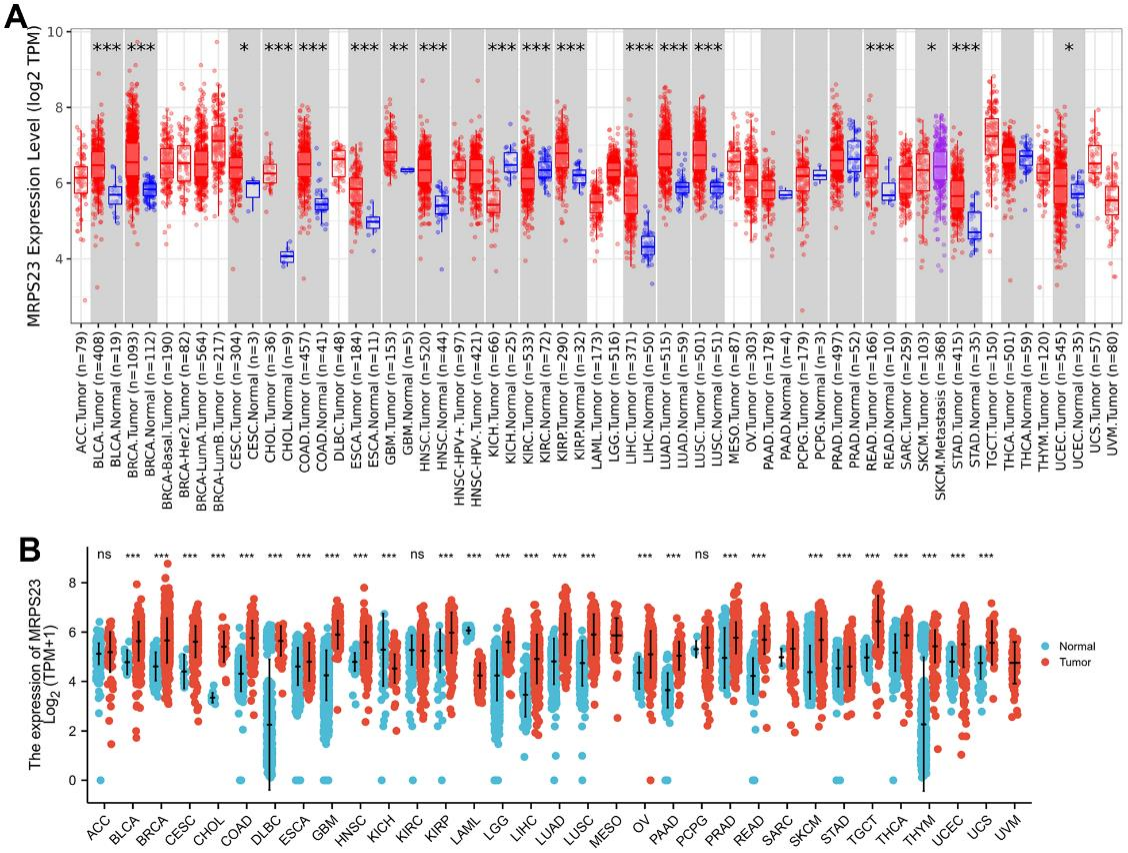
**MRPS23 expressed differentially between tumor and normal tissues.** (**A**) The MRPS23 expression in pan-cancer (TIMER database). (**B**) The MRPS23 expression in pan-cancer analysis by the TCGA/GTEx database. ns, p > 0.05, *p < 0.05, **p < 0.01, ***p < 0.001.

First, we excavated TIMER database to examine the MRPS23 expression level in pan-cancers, indicating that MRPS23 was increased in diverse human cancer, especially in gliomas (Figure 1A). Next, we combined the TCGA and GTEx cancer database, and found that MRPS23 was up-regulated in glioma than in paired adjacent normal tissues (Figure 1B).

### The prognosis values of MRPS23 in pan-cancer

**Figure 2:**
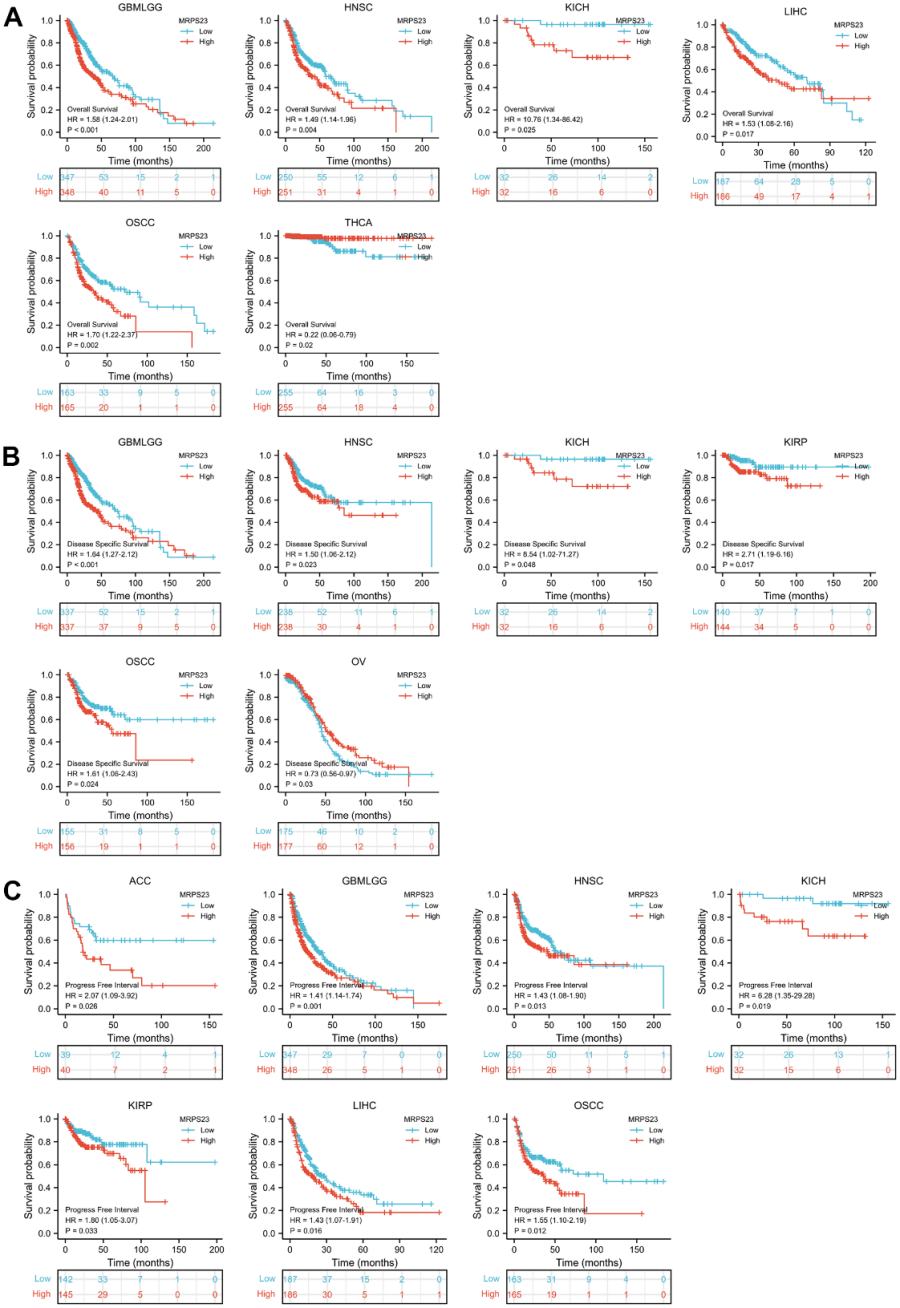
**MRPS23 expression correlated with the prognosis values of pan-cancer.** (**A**) The OS for MRPS23 in glioma, HNSC, KICH, LIHC, OSCC and THCA. (**B**) The DSS for MRPS23 in glioma, HNSC, LIHC, KICH, KIRP, OSCC and OV. (**C**) The PFI for MRPS23 in ACC, glioma, HNSC, KICH, KIRP, LIHC and OSCC.

We further explore the prognosis of MRPS23 in different tumor types. We found that increased MRPS23 expression correlated with poor OS for glioma, HNSC, KICH, LAML, LIHC, and oral squamous cell carcinoma (OSCC) (Figure 2A), poor DSS in glioma, HNSC, KICH, KIRP, and OSCC (Figure 2B), and poor PFI in adrenocortical carcinoma (ACC), glioma, HNSC, KICH, KIRP, LIHC, and OSCC (Figure 2C).

### MRPS23 could act as a potential diagnostic biomarker in pan-cancer

**Figure 3:**
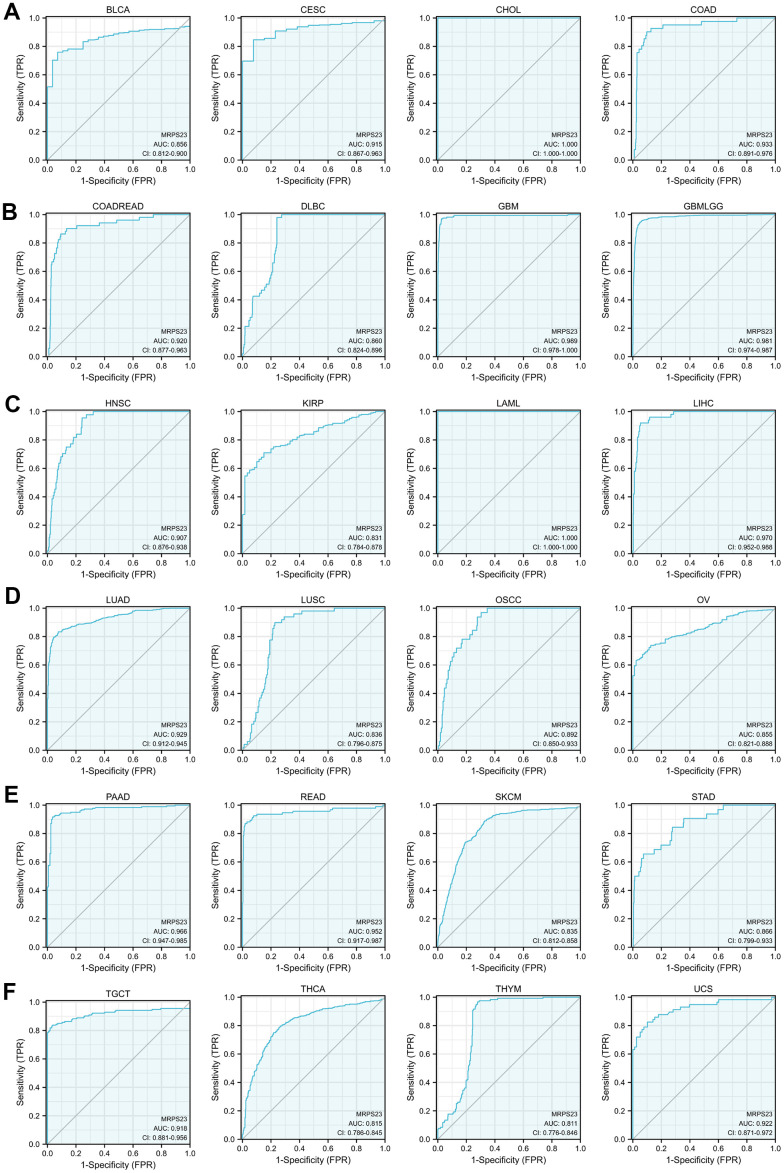
**MRPS23 may act as a potential biomarker in pan-cancer.** Predictive power for prognosis with MRPS23 expression by ROC curve analysis in BLCA, CESC, CHOL, and COAD (**A**); COADREAD, DLBC, GBM, and glioma (**B**); HNSC, KIRP, LAML, and LIHC (**C**); LUAD, LUSC, OSCC, and OV (**D**); PAAD, READ, SKCM, and STAD (**E**), TGCT, THCA, THYM, and UCS (**F**).

The ability of ROC curves to serve as diagnostic markers has been previously reported in a large number of literatures. We continue to analyze the ROC curve values of MRPS23 in different tumor types. Results showed that MRPS23 (AUC>0.75) for diagnosing BLCA, CESC, CHOL, COAD, LAML, colon adenocarcinoma/rectum adenocarcinoma esophageal carcinoma (COADREAD), GBM, THCA, glioma, HNSC, KIRP, LIHC, THYM, OSCC, OV, SKCM, LUAD, STAD, READ, PAAD, SKCM, DLBC, TGCT, LUSC and UCS (Figure 3A–3F).

### Gene mutation landscape of MRPS23

**Figure 4:**
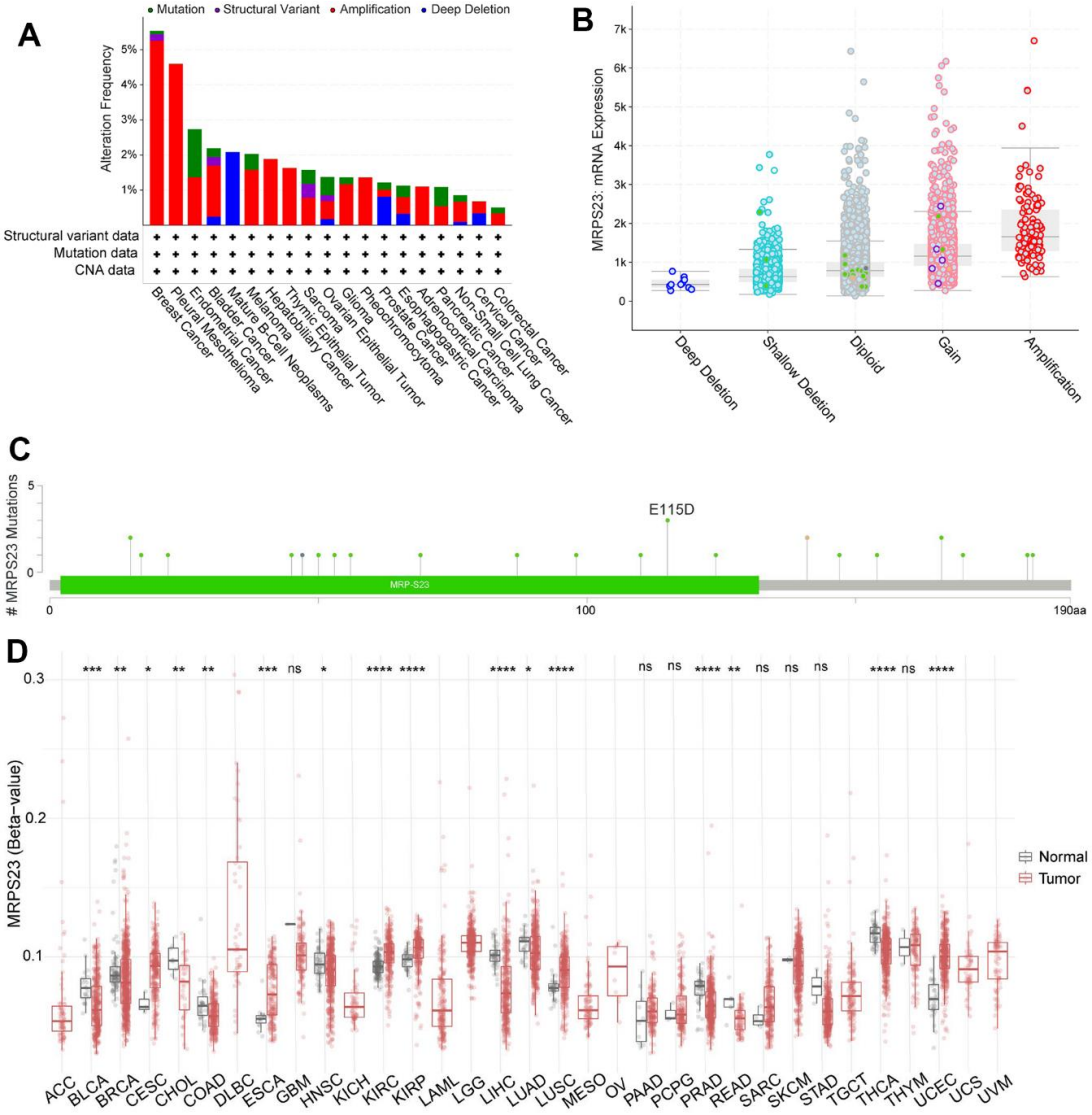
**Mutational analysis of MRPS23.** (**A**) Summary of mutation types of MRPS23 and the distribution among different cancers. (**B**) MRPS23 mutation frequency in pan-cancer (cBioPortal database). (**C**) Hot spots of mutation of MRPS23. (**D**) The correlation between MRPS23 methylation and expression.

**Figure 5:**
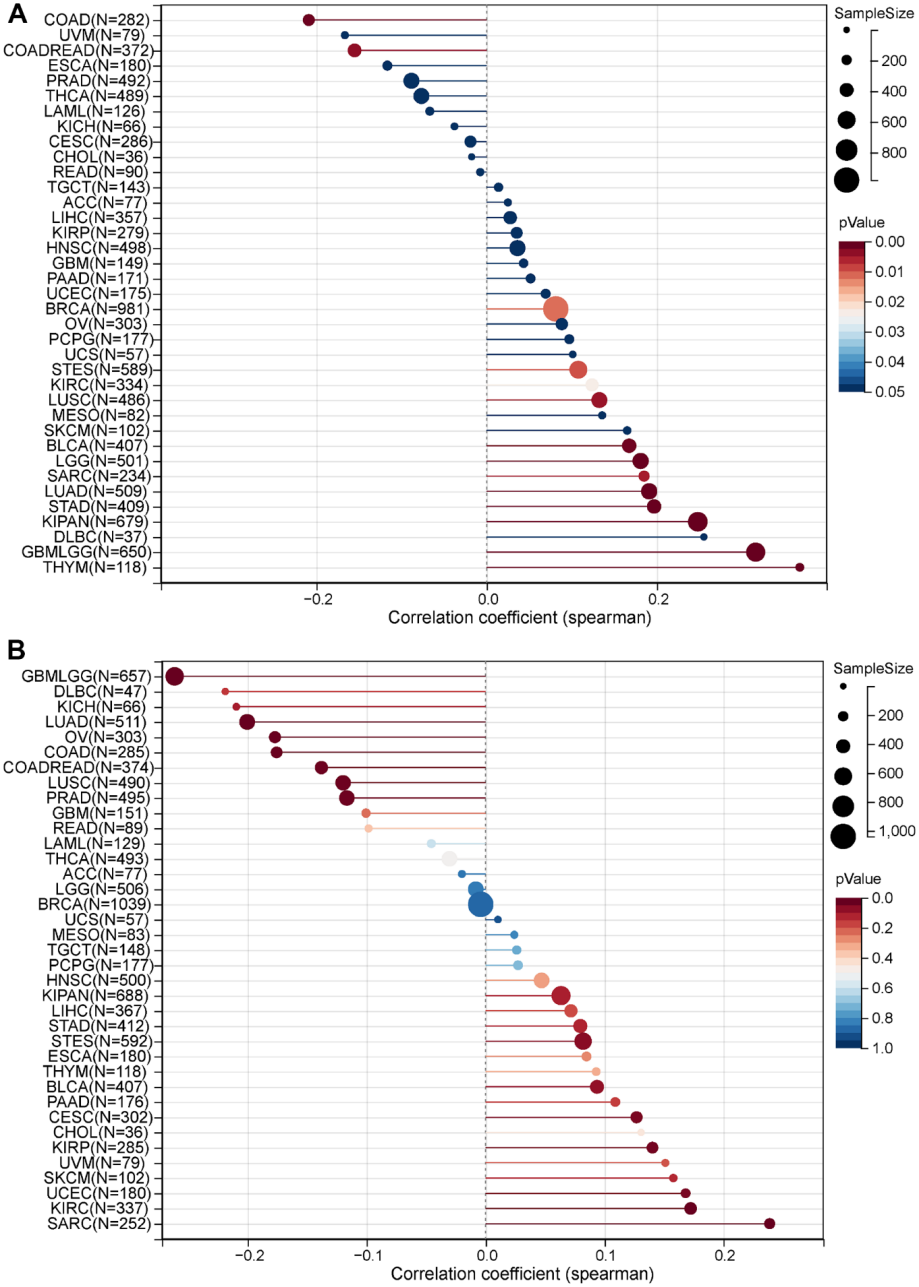
**Correlation between MRPS23 expression and TMB and MSI.** (**A**) Analysis of the correlation between MRPS23 and TMS in pan-cancer. (**B**) Analysis of the correlation between MRPS23 and MSI in pan-cancer.

We analyzed the MRPS23 mutational data. In cBioPortal, the mutation frequency was higher BRCA, pleural mesothelioma, endometrial cancer, BLCA, HNSC, and mature B-Cell neoplasms than in other types of cancer (Figure 4A). In addition, amplification was the most frequent alteration type (Figure 4B). MRPS23 has different mutation types in different tumors (Figure 4C). Moreover, DNA methylation analysis indicated that MRPS23 expression may be regulated by DNA methylation in BLCA, BRCA, CHOL, HNSC, LIHC, PRAD, READ, LUAD, and THCA (Figure 4D). All these results indicate that MRPS23 genetic alterations affect MRPS23 prognostic ability. MRPS23 had a positive relationship with TMB in LUAD, BRCA, glioma, LGG, STES, SARC, KIPAN, STAD, KIRC, LUSC, THYM, and BLCA (Figure 5A). MRPS23 had positive relationship with MSI in CESC, STES, SARC, KIRP, UCEC, and KIRC, and had negative relationship with glioma, LUAD, COAD, COADREAD, PRAD, LUSC, and OV (Figure 5B). Collectively, these results confirmed that MRPS23 can influence antitumor immunity.

### Immune cell infiltration of MRPS23

**Figure 6:**
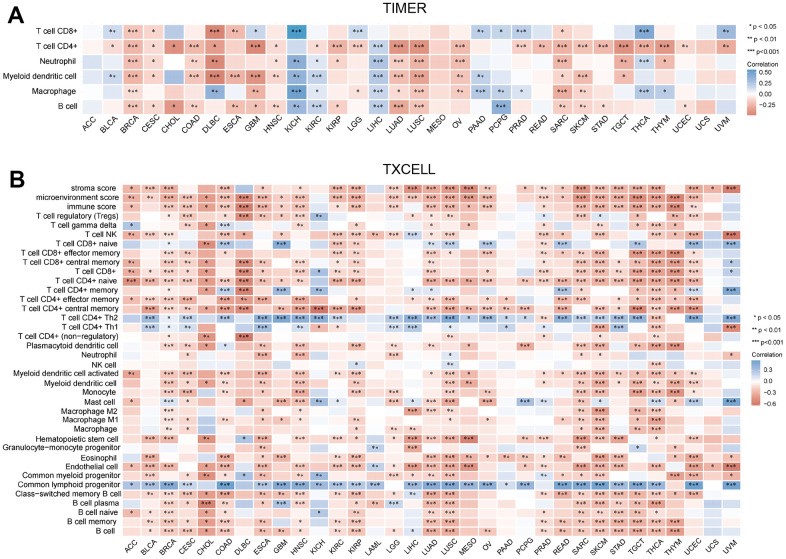
**Correlation between MRPS23 expression and immune infiltrates.** (**A**) Correlations between MRPS23 expression and the level of immune infiltration in 33 types of human cancer using TIMER. (**B**) Correlations between MRPS23 expression and the level of immune infiltration in 33 types of human cancer using XCELL. *p <0.05, **p < 0.01, ***p < 0.001.

Immune cells have an important role in tumor development [11]. TIMER database was used to reveal the relationship between the infiltration levels and CCDC50 expression in 32 types of cancers. In 30 kinds of cancers, the expression of MRPS23 was highly correlated with 6 major immune cells (Figure 6A). MRPS23 expression level had high correlation with the stroma score in 25 types human cancer, the microenvironment in 27 types cancer, the 38 types of immune cells in 32 types cancer, and the immune score in 24 types cancer (Figure 6B).

### Stem cell and RNA modification analysis of MRPS23

**Figure 7:**
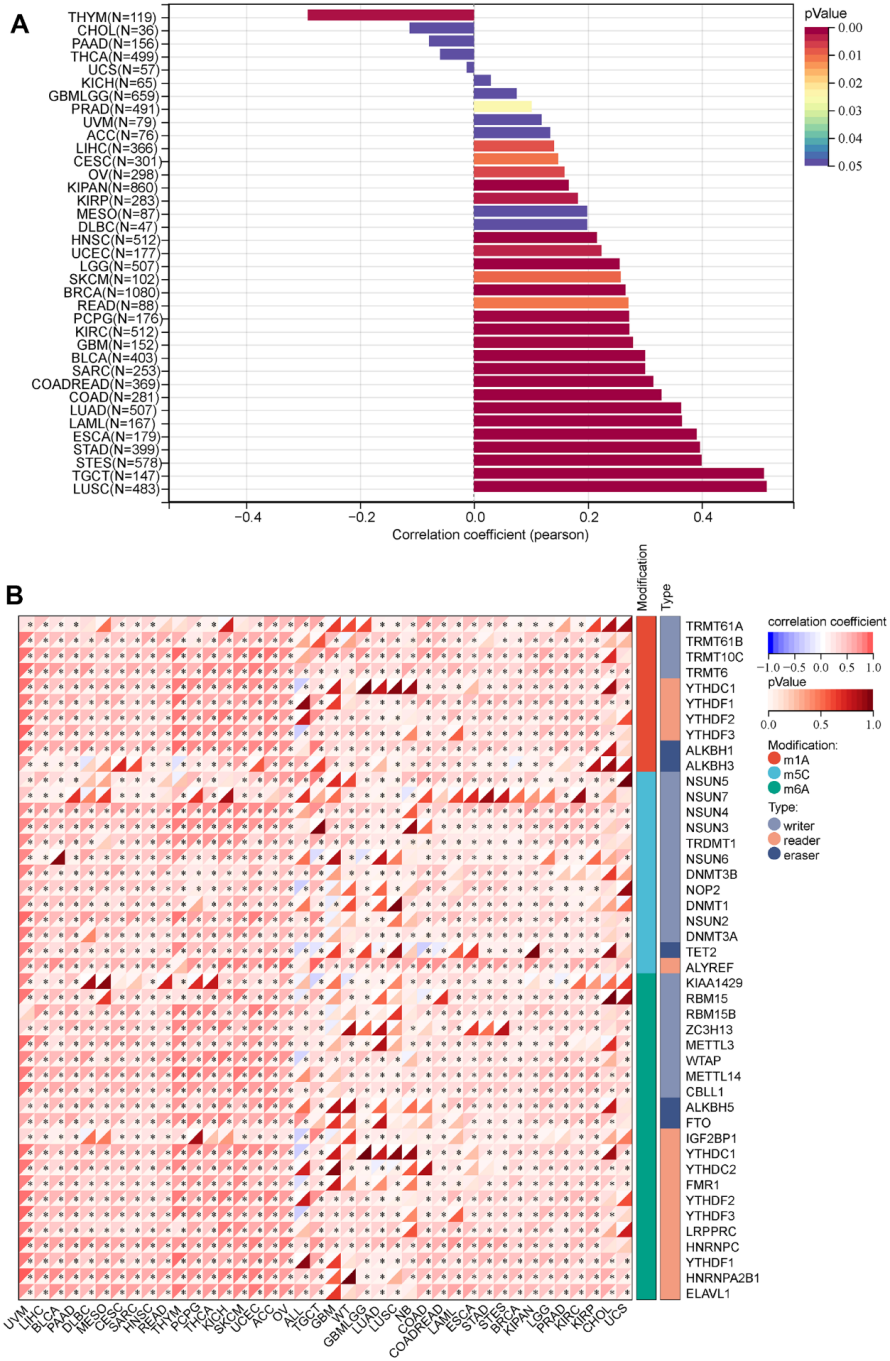
**Analysis of the correlation between the MRPS23 expression and stem cell and RNA modification in pan-cancer.** (**A**) Correlations between MRPS23 expression and the level of stem cell. (**B**) Correlations between MRPS23 expression and RNA modification.

It has a strong correlation between stem cells and malignant progression of cancer [12]. Here, we explore the relationship between MRPS23 expression level and stem cell. We found MRPS23 expression was positively correlated with CESC, LAML, BRCA, COADREAD, LIHC, STES, SARC, READ, OV, TGCT, PCPG, KIPAN, SKCM, and BLCA (Figure 7A).

RNA modification plays critical role in normal development and tumorigenesis [13]. The correlation between RNA modification regulators and MRPS23 in cancer was assessed using the TCGA TARGET GTEx. We revealed the relationship between MRPS23 expression and RNA modification regulators of pan-cancer. MRPS23 expression was markedly correlated with m1A, m5C and m6A in many types of cancers (Figure 7B).

### Interaction network of MRPS23 in pan-cancer

**Figure 8:**
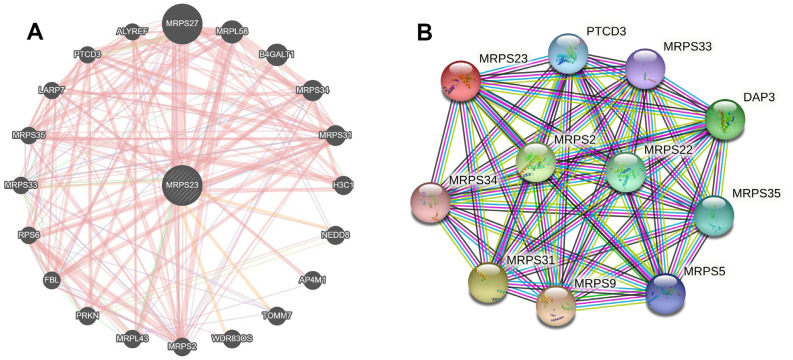
**Interaction network of MRPS23 at the gene and protein levels.** (**A**) Gene–gene interaction network of individual MRPS23 (GeneMANIA database). (**B**) Protein–protein interaction network of individual MRPS23 (STRING database).

The MRPS23 gene-gene interaction network is analyzed via GeneMANIA (Figure 8A). Next, we conducted MRPS23 interaction-protein network of the seed gene using STRING. As expected, several nodes and edges were obtained in the PPI network (Figure 8B).

### Clinical characteristics of MRPS23 in glioma

**Figure 9:**
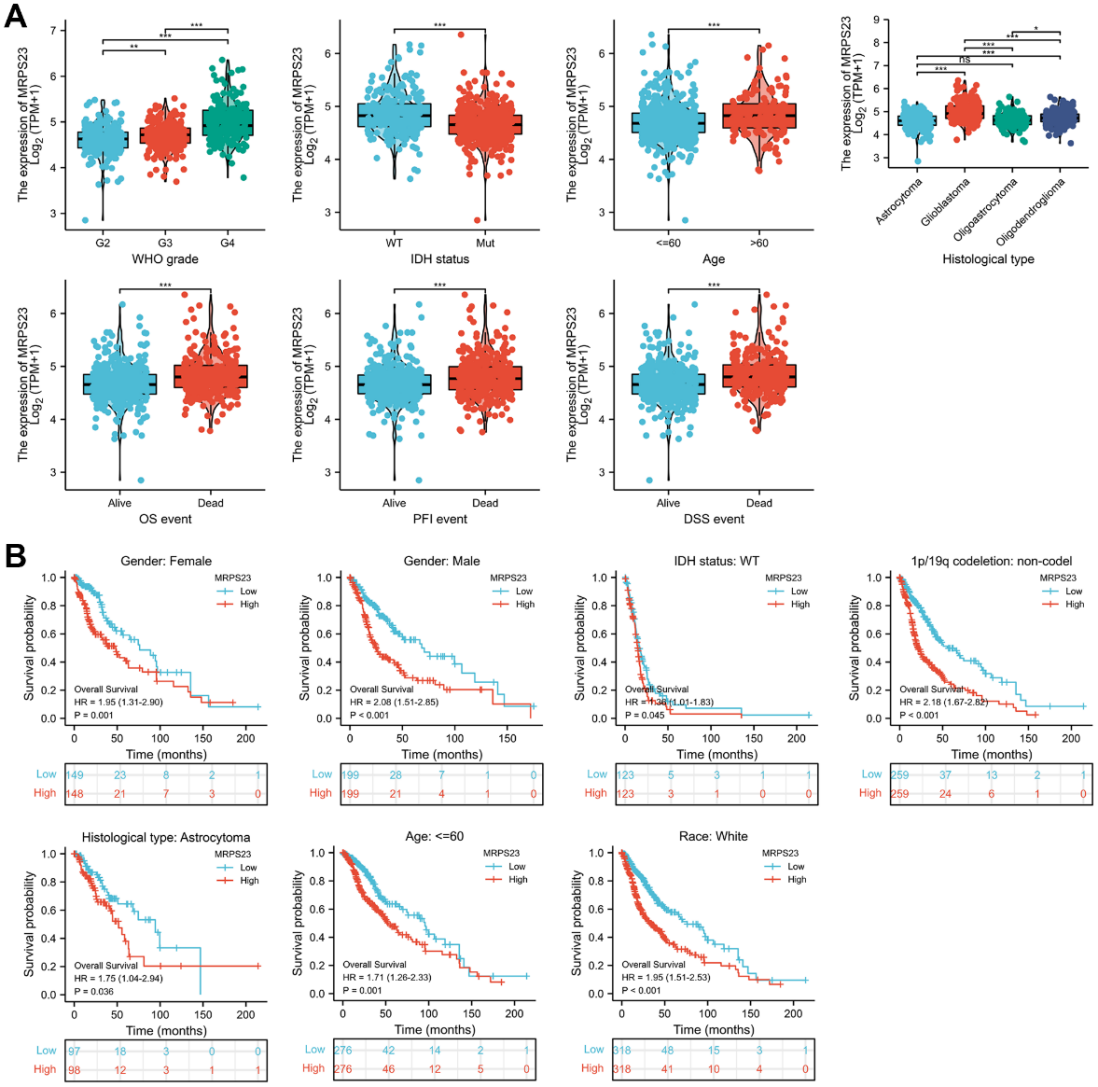
**MRPS23 is correlated with clinical characteristics and prognosis in glioma.** (**A**) The correlation between MRPS23 and clinical characteristics in glioma, including WHO grade, IDH status, age, histological type, OS event, DSS event, and PFI event. (**B**) The prognosis of MRPS23 in glioma.

**Figure 10:**
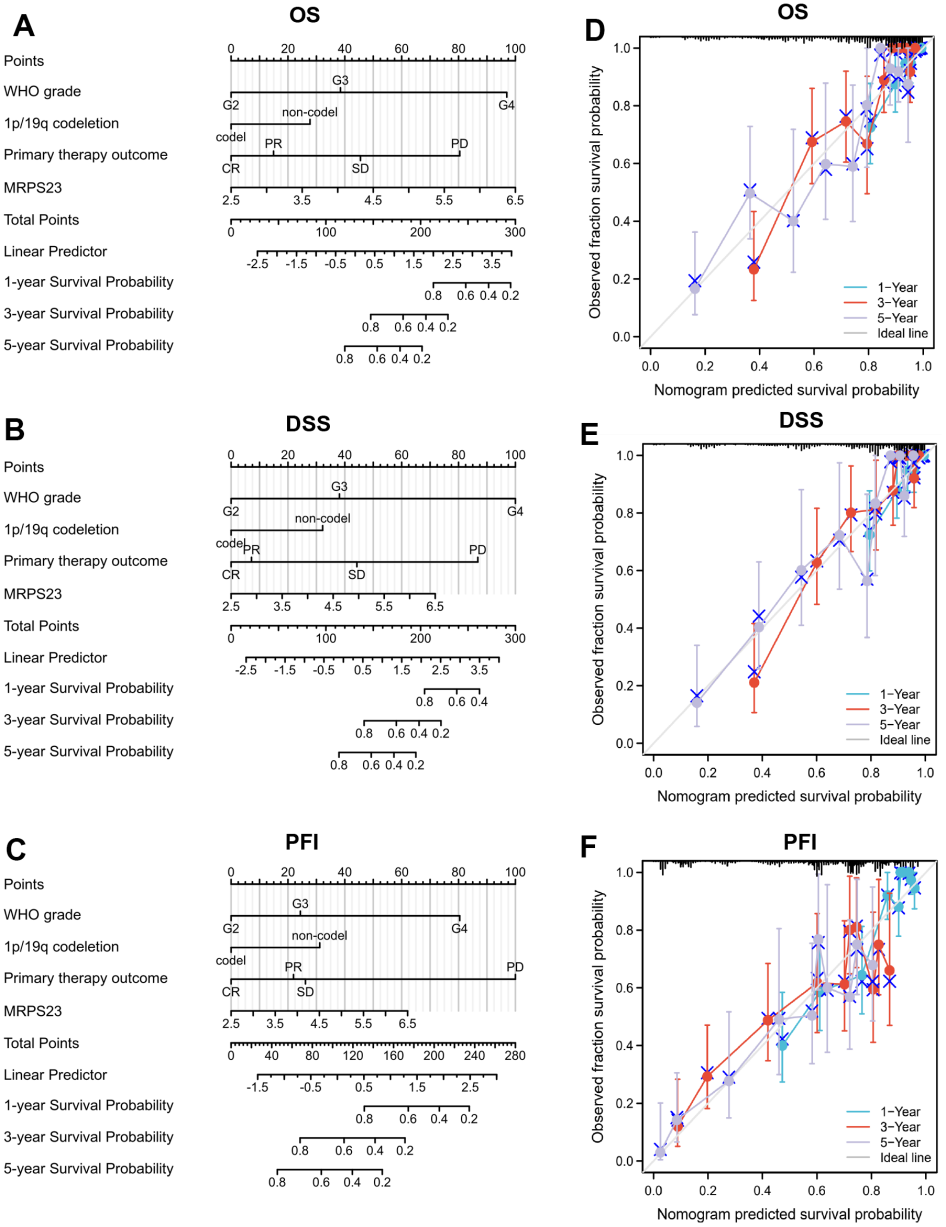
**Nomogram and calibration curve for predicting the probability of 1-, 3-, and 5-years OS for glioma patients.** (**A**–**C**) A nomogram integrates MRPS23 and other prognostic factors in glioma from TCGA data. (**D**–**F**) The calibration curve of the nomogram.

The relationship between MRPS23 expression level and glioma pathology was also explored. High MRPS23 expression level was correlated with adverse clinical features (Figure 9A and Table 1). Up-regulated MRPS23 had a worse OS in of glioma subgroups, including gender, IDH status (WT), 1p/19q codeletion (non-codel), histological type (astrocytoma), age (<=60), race (white) (Figure 9B). Cox regression analyses showed that WHO grade and MRPS23 expression level were associated with glioma prognosis (Tables 2–4). These confirmed that MRPS23 has an important role in glioma progression. A nomogram was contrasted to confirm MRPS23 as a glioma biomarker to predict OS, DSS, and PFI. As shown in (Figure 10A–10F), the calibration curves had predictions for the three nomograms for clinical outcomes (1-, 3-, and 5-years) (Figure 10A–10F). Therefore, it is expected to become a model to predict survival of glioma patients with MPRS23.

**Table 1:** MRPS23 expression associated with clinical pathological characteristics in glioma (logistic regression).

**Characteristic**	**Low expression of MRPS23**	**High expression of MRPS23**	**p**
n	348	348	
WHO grade, n (%)			< 0.001
G2	146 (23%)	78 (12.3%)	
G3	121 (19.1%)	122 (19.2%)	
G4	43 (6.8%)	125 (19.7%)	
IDH status, n (%)			< 0.001
WT	85 (12.4%)	161 (23.5%)	
Mut	259 (37.8%)	181 (26.4%)	
Race, n (%)			0.024
Asian	2 (0.3%)	11 (1.6%)	
Black or African American	14 (2%)	19 (2.8%)	
White	327 (47.9%)	310 (45.4%)	
Age, n (%)			< 0.001
<=60	300 (43.1%)	253 (36.4%)	
>60	48 (6.9%)	95 (13.6%)	
Histological type, n (%)			< 0.001
Astrocytoma	126 (18.1%)	69 (9.9%)	
Glioblastoma	43 (6.2%)	125 (18%)	
Oligoastrocytoma	84 (12.1%)	50 (7.2%)	
Oligodendroglioma	95 (13.6%)	104 (14.9%)	
OS event, n (%)			< 0.001
Alive	248 (35.6%)	176 (25.3%)	
Dead	100 (14.4%)	172 (24.7%)	
DSS event, n (%)			< 0.001
Alive	251 (37.2%)	180 (26.7%)	
Dead	89 (13.2%)	155 (23%)	
PFI event, n (%)			< 0.001
Alive	203 (29.2%)	147 (21.1%)	
Dead	145 (20.8%)	201 (28.9%)	
Age, median (IQR)	40 (31, 52.25)	52 (38, 62)	< 0.001

**Table 2:** Univariate and multivariate Cox regression analyses of different parameters on OS in glioma.

**Characteristics**	**Total (N)**	**Univariate analysis**		**Multivariate analysis**	
		**Hazard ratio (95% CI)**	**P-value**	**Hazard ratio (95% CI)**	**P-value**
WHO grade	634				
G2	223				
G3	243	2.999 (2.007-4.480)	<0.001	2.193 (1.406-3.421)	<0.001
G4	168	18.615 (12.460-27.812)	<0.001	7.812 (2.354-25.919)	<0.001
1p/19q codeletion	688				
codel	170				
non-codel	518	4.428 (2.885-6.799)	<0.001	1.492 (0.821-2.711)	0.190
Primary therapy outcome	461				
PD	112				
SD	147	0.440 (0.294-0.658)	<0.001	0.322 (0.199-0.522)	<0.001
PR	64	0.170 (0.074-0.391)	<0.001	0.172 (0.061-0.485)	<0.001
CR	138	0.133 (0.064-0.278)	<0.001	0.137 (0.064-0.291)	<0.001
Age	695				
<=60	552				
>60	143	4.668 (3.598-6.056)	<0.001	4.844 (2.955-7.940)	<0.001
Histological type	695				
Astrocytoma	195				
Glioblastoma	168	6.791 (4.932-9.352)	<0.001		
Oligoastrocytoma	134	0.657 (0.419-1.031)	0.068	0.990 (0.583-1.681)	0.970
Oligodendroglioma	198	0.580 (0.395-0.853)	0.006	0.620 (0.355-1.083)	0.093
MRPS23	695	2.639 (1.975-3.525)	<0.001	1.908 (0.903-4.031)	0.090

**Table 3:** Univariate and multivariate Cox regression analyses of different parameters on DSS in glioma.

**Characteristics**	**Total (N)**	**Univariate analysis**		**Multivariate analysis**	
		**Hazard ratio (95% CI)**	**P-value**	**Hazard ratio (95% CI)**	**P-value**
WHO grade	614				
G2	220				
G3	239	3.098 (2.023-4.744)	<0.001	2.158 (1.356-3.435)	0.001
G4	155	19.243 (12.572-29.455)	<0.001	8.026 (2.388-26.971)	<0.001
1p/19q codeletion	668				
codel	169				
non-codel	499	4.987 (3.117-7.978)	<0.001	1.691 (0.894-3.198)	0.106
Primary therapy outcome	457				
PD	111				
SD	144	0.372 (0.242-0.572)	<0.001	0.276 (0.164-0.463)	<0.001
PR	64	0.138 (0.056-0.343)	<0.001	0.124 (0.038-0.405)	<0.001
CR	138	0.116 (0.053-0.252)	<0.001	0.120 (0.054-0.266)	<0.001
Age	674				
<=60	541				
>60	133	4.500 (3.409-5.940)	<0.001	4.419 (2.625-7.439)	<0.001
Histological type	674				
Astrocytoma	192				
Glioblastoma	155	6.602 (4.739-9.197)	<0.001		
Oligoastrocytoma	132	0.604 (0.374-0.975)	0.039	0.971 (0.559-1.685)	0.917
Oligodendroglioma	195	0.543 (0.363-0.813)	0.003	0.629 (0.353-1.122)	0.116
MRPS23	674	2.696 (1.995-3.644)	<0.001	1.665 (0.777-3.568)	0.190

**Table 4:** Univariate and multivariate Cox regression analyses of different parameters on PFI in glioma.

**Characteristics**	**Total (N)**	**Univariate analysis**		**Multivariate analysis**	
		**Hazard ratio (95% CI)**	**P-value**	**Hazard ratio (95% CI)**	**P-value**
WHO grade	634				
G2	223				
G3	243	1.616 (1.197-2.182)	0.002	1.377 (0.976-1.943)	0.068
G4	168	7.865 (5.808-10.649)	<0.001	2.665 (0.894-7.948)	0.079
1p/19q codeletion	688				
codel	170	Reference			
non-codel	518	3.373 (2.438-4.666)	<0.001	1.535 (0.980-2.404)	0.061
Primary therapy outcome	461				
PD	112				
SD	147	0.253 (0.178-0.358)	<0.001	0.225 (0.152-0.332)	<0.001
PR	64	0.226 (0.137-0.372)	<0.001	0.209 (0.113-0.388)	<0.001
CR	138	0.160 (0.104-0.246)	<0.001	0.147 (0.092-0.235)	<0.001
Age	695				
<=60	552				
>60	143	2.873 (2.268-3.640)	<0.001	2.614 (1.739-3.928)	<0.001
Histological type	695				
Astrocytoma	195				
Glioblastoma	168	4.416 (3.353-5.816)	<0.001		
Oligoastrocytoma	134	0.578 (0.401-0.832)	0.003	0.762 (0.490-1.183)	0.226
Oligodendroglioma	198	0.638 (0.469-0.868)	0.004	0.658 (0.420-1.031)	0.068
MRPS23	695	2.362 (1.763-3.165)	<0.001	1.545 (0.894-2.672)	0.119

### Biological functions of MRPS23 in glioma

**Figure 11:**
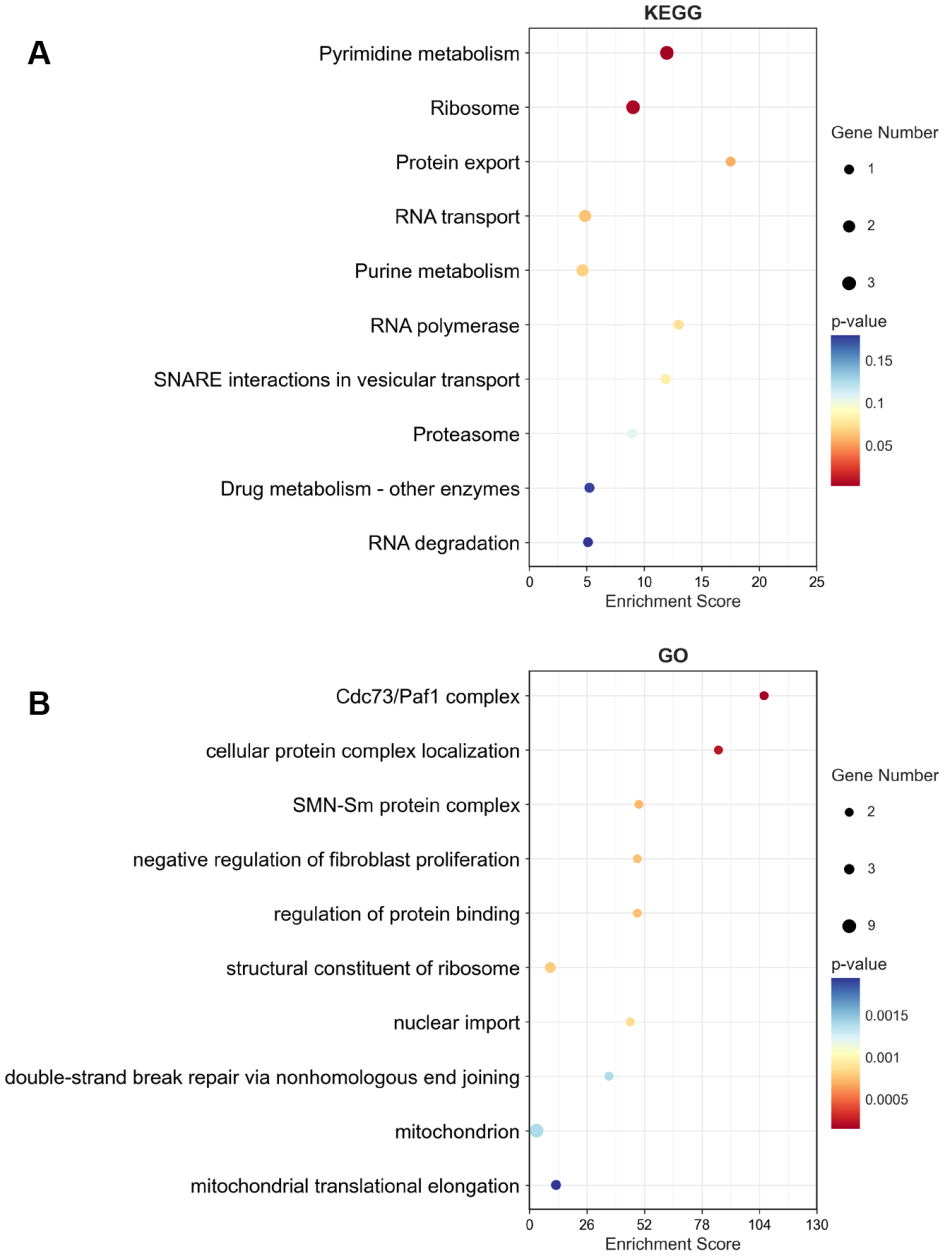
**KEGG and GO enrichment of top 50 similar genes related to MRPS23 in glioma.** The KEGG term (**A**) and GO term (**B**) of MRPS23 analysis by using top 50 similar genes related to it in glioma.

To analyze GO and KEGG enrichment, we discovered the top 50 similar genes related to MRPS23 in glioma using the GEPIA database. GO analysis results confirmed that MRPS23 mainly participated in Cdc73/Paf1 complex, cellular protein complex localization, SMN-Sm protein complex, negative regulation of fibroblast proliferation, regulation of protein binding, structural constituent of ribosome, nuclear import, mitochondrion, and mitochondrial translational elongation (Figure 11A, 11B). In summary, MRPS23 plays a crucial role in regulating glioma malignant progression.

### Down-regulation of MRPS23 suppresses the malignant phenotype of glioma

**Figure 12:**
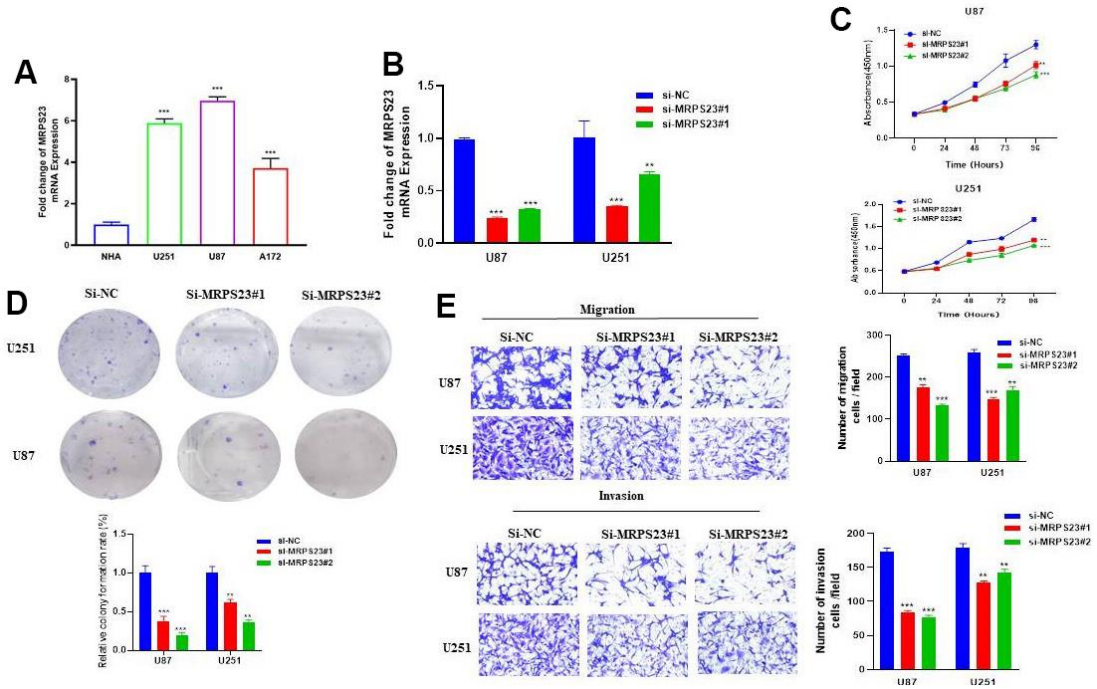
**MRPS23 promotes the proliferation, migration, and invasion of glioma cells.** (**A**) The expression of MRPS23 in NHA cell and glioma cell lines was examined via qRT-PCR assay. (**B**) The establishment of MRPS23 knockdown cell lines in U87 and U251 was verified via qRT-PCR assay. (**C**, **D**) The knockdown of MRPS23 dramatically inhibits the proliferation of U87 and U251 cells, examined via cell counting kit-8 assay and colony formation assay. (**E**, **F**) The knockdown of MRPS23 dramatically inhibits the migration and invasion abilities of U87 and U251 cells. Data are presented as the mean ± SD of three independent experiments. **p < 0.01, ***p < 0.001.

The biological function of MRPS23 in glioma progression has been further explored and verified. We found that compared with normal astrocytes (NHA), MRPS23 was upregulated in glioma cell lines and glioma tissues (Figure 12A and Supplementary Figure 1A). We used CGGA database analysis and found that high expression of MRPS23 was associated with adverse clinical characteristics and poor prognosis in glioma patients (Supplementary Figure 1B–1F). We further used siRNA of MRPS23 to down-regulate its expression in U87 and U251 cells. The knockdown efficacy was verified via qRT-PCR by using cell lines expressing a negative control (Figure 12B). As expected, MRPS23 knockdown inhibited the proliferation of U87 and U251 cells (Figure 12C, 12D). The migration ability of glioma cells was significantly inhibited after MRPS23 gene knockdown (Figure 12E, 12F). We also found that the expression level of MRPS23 was significantly positively correlated with the expression of m6A modification-related genes (Supplementary Figure 1G–1I). These results support an oncogenic role of MRPS23 in glioma.

## DISCUSSION

Pan-cancer analysis is crucial for the study of revealing tumor molecular markers. MRPS23 has been demonstrated to be associated with the development of BRCA, OS and LIHC [14, 15]. Currently, no studies have tested whether MRPS23 is associated with cancer prognosis. In this research, MRPS23 expression was higher in BRCA, CHOL, BLCA, COAD, DLBC, CESC, LIHC, GBM, THYM, HNSC, KIRP, ESCA, LUAD, LGG, PRAD, THCA, READ, STAD, SKCM, OV, TGCT, PAAD, LUSC, UCEC, and UCS. In addition, our result indicated high MRPS23 expression correlated with poor OS for glioma, HNSC, KICH, LIHC, OSCC, and THCA, poor DSS in glioma, HNSC, KICH, KIRP, OSCC, and OV, and poor PFI in ACC, glioma, HNSC, KICH, KIRP, LIHC, and OSCC.

ROC curve analysis indicated that MRPS23 is expected to be used as a biomarker for the diagnosis of pan-cancer with high specificity and sensitivity. Additionally, our team ensured the relationship between MRPS23 and mutation, alteration frequencies were 5.26%, 4.6%, 3.02%, and 2.19% in BRCA, pleural mesothelioma, endometrial cancer, BLCA, HNSC, and mature B-Cell neoplasms. Moreover, MRPS23 expression was negatively correlated to DNA methylation in BLCA, BRCA, PRAD, CHOL, HNSC, LUAD, THCA, READ, and LIHC. TMB and MSI are a biomarker for cancer immune checkpoint inhibitors [16, 17]. It has been reported that MRPS23 could inhibit NF-κB signaling pathway in OS cell [18]. Our research also indicated MRPS23 expression level is in relation to the stem cell, infiltration of immune cells, m1A, m5C and m6A in human cancer. Further, MRPS23 was strongly associated with clinical subgroups and OS of LIHC.

Glioma is a multiple malignant tumor, which is a serious threat to human health [19]. We found that decreased MRPS23 expression levels were critical independent prognostic factors, and directly associated with better OS, DSS, and PFS outcomes. GO enrichment results indicated that these genes were mainly involved in Cdc73/Paf1 complex, cellular protein complex localization, SMN-Sm protein complex, negative regulation of fibroblast proliferation, regulation of protein binding, structural constituent of ribosome, nuclear import, mitochondrion, and mitochondrial translational elongation. This study broadens our horizon of MRPS23 in glioma, but there are still some inconsistencies. Firstly, we have only investigated the relationship between MRPS23 and RNA modification, and immune infiltration, there is no experimental date to confirm the function of MRPS23 in the regulation of immune infiltration. Secondly, the molecular mechanism of MRPS23 upregulation in glioma remains unstudied.

## CONCLUSIONS

To sum up, we revealed that MRPS23 was upregulated in glioma cancer cell lines, and knockdown of MRPS23 inhibited the proliferation, migration, and invasion of glioma cancer cells. MRPS23 can be used as a molecular predictor of cancer patients, and can be used as a clinical diagnosis and treatment target of glioma.

## Supplementary Material

Supplementary Figure 1
