# Supplementary material for: MRPS23 is a novel prognostic biomarker and promotes glioma progression
Source: Aging (Albany NY). 2024 Jan 31;16(3):2457–74. doi: 10.18632/aging.205493 (PMC10911364; doi:10.18632/aging.205493)
Supplement: Supplementary Figure 1 [file aging-16-205493-s001.pdf]

SUPPLEMENTARY FIGURE

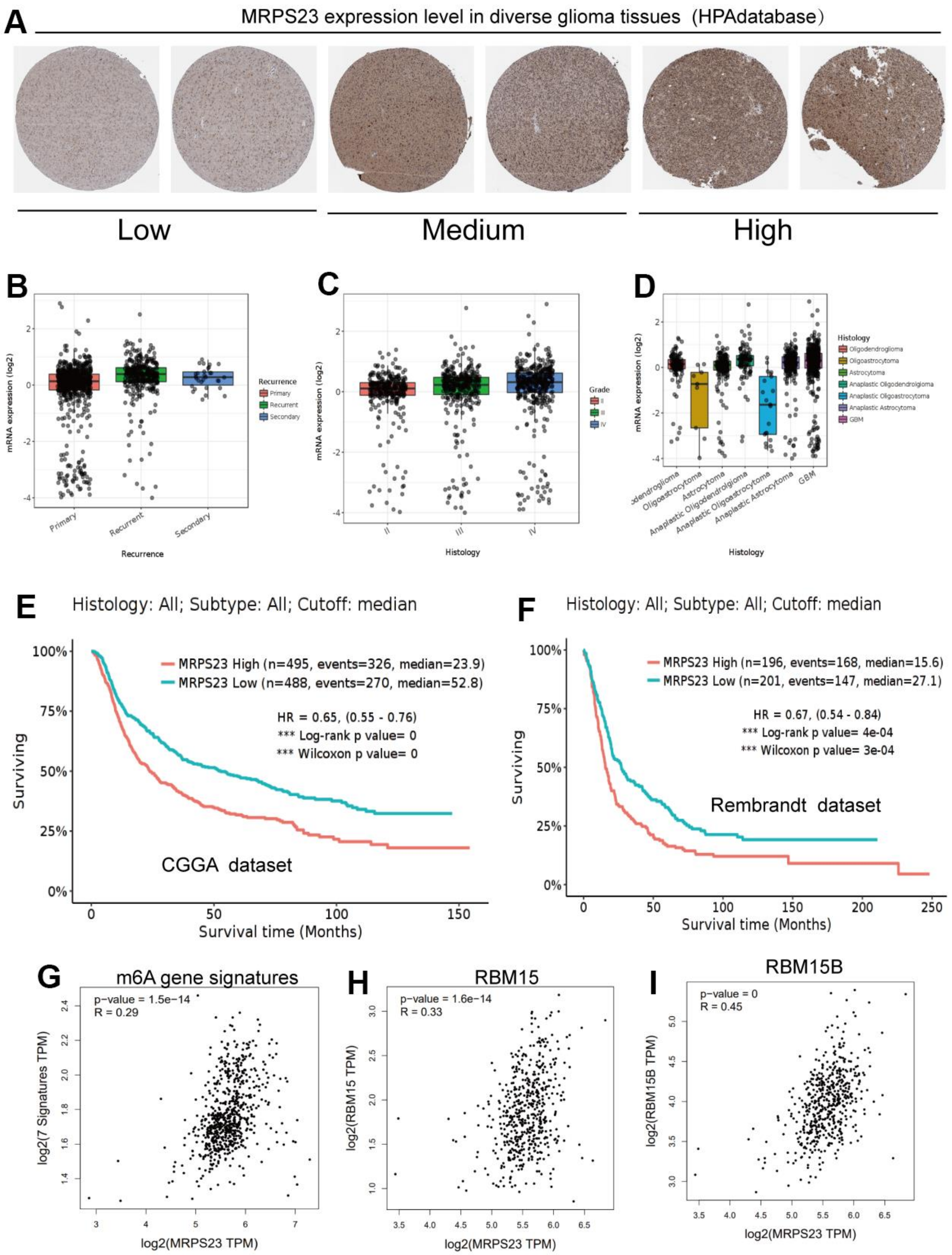

Supplementary Figure 1. MRPS23 was highly expressed in glioma and correlated with adverse clinical outcomes in glioma patients. (**A**) MRPS23 is highly expressed in glioma tissues examined by HPA database. (**B**–**D**) High expression of MRPS23 correlated with adverse clinical features in glioma patients, examined by CGGA dataset. (**E**, **F**) High expression of MRPS23 correlated with adverse clinical outcomes in glioma patients, examined by CGGA and Rembrandt dataset. (**G**–**I**) High expression of MRPS23 positively correlated with m6A methylation regulator in glioma patients, examined by CGGA and Rembrandt dataset.
